# Manual of Operative Surgery

**Published:** 1898-12

**Authors:** 


**Manual of Operative Surgery.** By H. J. Waring, M.S., M.B. Pp. xxvi., 661. Edinburgh: Young J. Pentland. 1898.

This is intended to serve as a complete handbook to students; but many subjects are dealt with, such as craniectomy, pylorectomy, draining the lateral ventricles and hepatectomy, which it is the lot of few to witness and still fewer to perform. The book is well illustrated by 400 illustrations; but fig. 98 on p. 110 does not correspond to the text.

The operations are described under (1) Indications, (2) Position of patient, operator and assistants, (3) Instruments, (4) Stages, (5) After-treatment. The descriptions of the operations are very perfect, and attention is drawn to important points connected therewith. Altogether the book may be strongly recommended as an excellent guide in a course of Operative Surgery.

